# Increased serum total bilirubin-albumin ratio was associated with bilirubin encephalopathy in neonates

**DOI:** 10.1042/BSR20192152

**Published:** 2020-01-31

**Authors:** Yan Wang, Guangyao Sheng, Lina Shi, Xiuyong Cheng

**Affiliations:** 1 Department of Pediatrics, The First Affiliated Hospital of Zhengzhou University, Zhengzhou, Henan Province 450052, China;; 2 Henan Medical College, Zhengzhou, Henan Province 451191, China; 3 Department of Neonatology,The Affiliated Hospital of Zhengzhou University, Zhengzhou, Henan Province 450052, China

**Keywords:** bilirubin encephalopathy, bilirubin-albumin ratio, total bilirubin

## Abstract

We performed the present study to summarize the recent epidemiological characteristics of bilirubin encephalopathy and assess the role of total bilirubin-albumin ratio in the bilirubin encephalopathy. We retrospectively collected clinical data of 669 neonates with hyperbilirubinemia from the First Affiliated Hospital of Zhengzhou University between January 2015 and July 2018, including 153 neonates belonged to bilirubin encephalopathy and 516 ones were treated as control group. Compared with the control group, those with bilirubin encephalopathy have higher bilirubin-albumin ratio (13.8 ± 3.6 vs. 10.6 ± 2.5, *P*=0.000). The direct bilirubin and indirect bilirubin level were higher in the case group than that in the control group (*P*=0.000). On the contrary, the hemoglobin level was lower in the case group than that in the control group (*P*=0.004). There were no significant differences in gestational age (*P*=0.510), gender rate (*P*=0.313), maternal gestational diabetes ratio (*P*=0.071), natural childbirth ratio (*P*=0.686), and meconium delay (*P*=0.091). The results from univariate regression indicated the total bilirubin/albumin ratio was positively associated with bilirubin encephalopathy (odds ratio (OR) = 1.67, 95% confidence interval (CI): 1.59–3.14). The total bilirubin, direct bilirubin, and indirect bilirubin were also related to encephalopathy. After adjusting some potential cofounding factors, the total bilirubin-albumin was still associated with bilirubin encephalopathy. The higher total bilirubin-albumin ratio increased the risk of bilirubin encephalopathy by 23% (OR = 1.23, 95% CI: 1.16–2.48). Our results indicated that the bilirubin-albumin ratio is associated with bilirubin encephalopathy in neonates, and could be a potential predictor.

## Introduction

Clinical jaundice is present in the majority of the newborns. Although most jaundice are benign, there are still some infants suffering from bilirubin encephalopathy due to the extremely high serum bilirubin level and the development of bilirubin-induced neurologic injury [1,2]. Bilirubin encephalopathy was reported worldwide. It can be classified as acute bilirubin encephalopathy and chronic bilirubin encephalopathy [3]. Although we have paid more attention to the disease and the medical techniques are constantly improved, bilirubin encephalopathy still occurs frequently. The prediction and diagnosis of bilirubin encephalopathy are dependent on the assessment of clinical symptoms, total serum bilirubin, bilirubin to albumin ratio, auditory brainstem response, and magnetic resonance imaging [4,5]. In China, some people used the neonatal behavioral neurological assessment to assess the clinical symptoms, but it was complex and less specific in predicting bilirubin encephalopathy. Bilirubin-induced neurological dysfunction score was easy to operate and have been used by many international researchers [6]. The total serum bilirubin and bilirubin to albumin ratio were the most commonly used laboratory examinations to predict bilirubin encephalopathy, but their usefulness is controversial. Until now, none of these methods can be a perfect prediction of bilirubin encephalopathy and clinical management relies on the comprehensive assessment of clinical symptoms and the laboratory or imaging examinations [7,8]. The epidemiological study can provide basis for clinical management. Many years have passed since the last multicenter epidemiological investigation. Whether the recent epidemiological characteristic is different from the past is still unknown. And more researches are needed. We performed the present study to summarize the recent epidemiological characteristics of bilirubin encephalopathy and assess the role of total bilirubin-albumin ratio in the bilirubin encephalopathy.

## Materials and methods

### Study population

We retrospectively collected data of neonates with hyperbilirubinemia from The First Affiliated Hospital of Zhengzhou University between January 2015 and July 2018. Criteria for inclusion: (1) neonates were diagnosed with hyperbilirubinemia; (2) 37 gestational age: 37–42 weeks; (3) hyperbilirubinemia was diagnosed within 7 days. Criteria for exclusion: Neonates died within 12 h after birth; neonates with incomplete data and information; Those with intracranial infection, congenital malformation (chromosome abnormality), and family history of deafness. Severe hyperbilirubinemia: total serum bilirubinemia (TSB) > 342 μmol/l; very severe hyperbilirubinemia > 427 μmol/l; bilirubin encephalopathy: Besides TSB > 342 [9], There were typical signs and symptoms of nervous system, such as lethargy, convulsion, dystonia, and asthenia, etc. Head magnetic resonance imaging (MRI) showed characteristic changes of T1- and T2-weighted images of bilateral globes pallidus with high signal symmetry. Brain stem auditory evoked potential (BAEP) suggests high-frequency hearing loss, which is diagnosed according to severe hyperbilirubinemia and clinical manifestations. The present study was approved by the Ethics Committee of The First Affiliated Hospital of Zhengzhou University. The research was carried out in accordance with the World Medical Association Declaration of Helsinki, and all subjects provided written informed consent.

### Data collection

The general information included gestational age (week), admission age (h), gender (male/female), birth weight (g), maternal gestational diabetes, production type (natural childbirth vs. caesarean), meconium delay, and birth asphyxia. These data were collected through medical records. Other symptoms including ABO hemolysis, RH hemolysis, glucose 6 phosphate dehydrogenase (G6PD) deficiency, hypoglycemia, infectious, metabolic acidosis, cranial hematoma/hemorrhage were also collected. Serum total bilirubin, direct bilirubin, and indirect bilirubin levels were measured by the vanadate oxidation method using automatic biochemical analyzer. Hematologic test (albumin and hemoglobin) was measured using an automated hematology analyzer (Bayer Diagnostics, Newbury, Berkshire, U.K.).

In children with hyperbilirubinemia, there are neurological symptoms and signs, including lethargy, altered crying, abnormal muscular tone, opisthotonos, and convulsion. MRI of cephalalgia suggested that T1 or T2 symmetry hypersignals existed in bilateral globous pallidum. Listen for abnormalities in the tests (automated auditory brainstem response). G6PD deficiency was defined as the serum G6PD was lower than the lower limit (normal: 11.8–30 µ/g Hb) [10]. Infectious diseases include pneumonia, infection, urinary tract infection etc. Sepsis was defined as positive blood culture, or negative blood culture with changes in clinical manifestations and laboratory indicators, and received full course of anti-infection therapy. Hemolysis caused by ABO or Rh hemolysis or other incompatibility of maternal and infant blood groups is confirmed by neonatal hemolysis screening test. Hemolytic screening tests include blood group identification of parents and children, direct Coombs test, antibody release test, and free antibody test. Hypoglycemia was defined when the glucose level was less than 2.2 mmol/l [11].

### Statistical analysis

Study population were divided into bilirubin encephalopathy and hyperbilirubinemia group. Continuous data were expressed as mean ± standard deviation or median (minimum, maximum) according to the normality of data distribution. The independent sample *t* test or non-parameter test was used between case group and control group. The normality test was performed by Kolmogorov–Smirnov method. The category data were expressed using the count and percent. The Chi-square test was used to compare the differences between two group. We used the univariate and multivariate logistic (gestational age, admission age, gender, birth weight, maternal gestational diabetes, natural childbirth, meconium delay, premature sepsis, ABO hemolysis, RH hemolysis, G6PD deficiency, hypoglycemia, infectious, metabolic acidosis, cranial hematoma/hemorrhage, total bilirubin, total protein, albumin, total bilirubin/albumin, and hemoglobin) regression to explore the relationship between bilirubin-albumin ratio and brain injury in neonates. All analyses were performed using the SPSS 20.0 and GraphPad Prism 5.0. *P*<0.05 was considered statistically significant.

## Results

### General characteristics of study population

**Figure 1:**
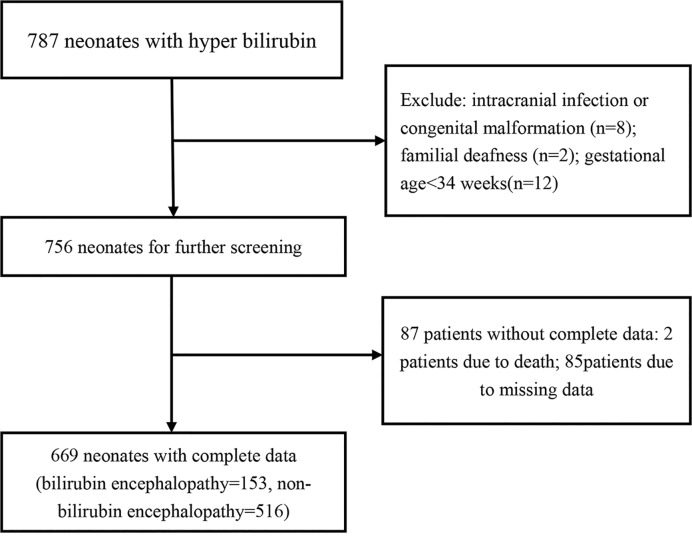
The selection flow chart of study population

Initially, we identified 787 neonates with hyper bilirubin. Eight neonates with intracranial infection/congenital malformation, two ones with family deafness, and twelve ones with gestational age < 34 weeks were excluded. Besides, eighty-seven patients, including two deaths and eighty-five ones with missing data were also excluded. Finally, 669 neonates with complete data were included in the analysis. A total of 153 neonates belonged to bilirubin encephalopathy (22.9%) and 516 ones were treated as control group (77.1%). The screening flow of study patients were presented in Figure 1. All of these participants, 61.8% (*n*=414) were boys and 38.2% of them were girls. The mean gestational age was 38.7 weeks. A total of 511 neonates were born by natural childbirth way (76.4%). The mean birth weight was 3243.1 g. Of all them, 20.9% of mother of neonates have gestational diabetes (*n*=140). The admission age was 103.6 h.

### Comparisons of characteristic between bilirubin encephalopathy and control group

**Figure 2:**
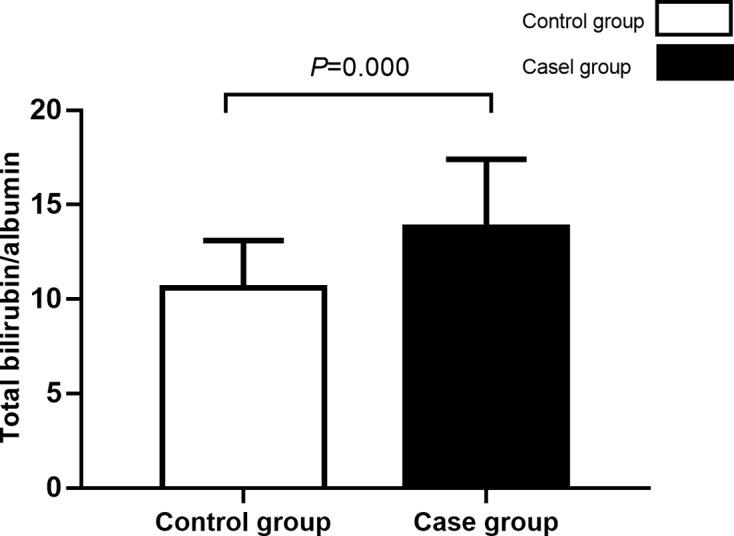
Comparisons of bilirubin albumin ratio between case group and control group

The Table 1 presented the results of clinical parameters between bilirubin encephalopathy group and control group. As we can see, there were no significant differences in gestational age (*P*=0.510), gender rate (*P*=0.313), maternal gestational diabetes ratio (*P*=0.071), natural childbirth ratio (*P*=0.686), and meconium delay (*P*=0.091). Compared with control group, neonates with bilirubin encephalopathy tend to have longer admission age (114.2 ± 34.8 vs. 100.5 ± 42.3, *P*=0.000), higher premature sepsis (37.9 vs. 21.9%, *P*=0.000), RH hemolysis (9.8 vs. 4.8%, *P*=0.037), G6PD deficiency (22.9 vs. 7.0%, *P*=0.000), and hypoglycemia ratio (6.5 vs. 2.3%, *P*=0.010). No significant differences were observed in ABO hemolysis (*P*=0.159), infectious (*P*=0.991), metabolic acidosis (*P*=1.825), cranial hematoma/hemorrhage (*P*=0.784). However, the direct bilirubin and indirect bilirubin levels were higher in the case group than that in the control group (*P*=0.000). The total bilirubin-albumin ratio was higher in the case group than that in the control group (13.8 ± 3.6 vs. 10.6 ± 2.5, *P*=0.000, Figure 2). On the contrary, the hemoglobin level was lower in the case group than that in the control group (*P*=0.004).

**Table 1:** Comparison of general characteristic between two groups

Factors	Case group (*n*=153)	Control group (*n*=516)	t/χ^2^	*P*-value
Gestational age, week	38.7 ± 1.8	38.8 ± 1.6	−0.659	0.510
Admission age, h	114.2 ± 34.8	100.5 ± 42.3	3.365	0.000
Gender (male/female)	100/53	314/202	1.016	0.313
Birth weight (g)	3168.6 ± 406.2	3265.2 ± 389.4	2.668	0.008
Maternal gestational diabetes, *n*	40 (26.1)	100 (19.4%)	3.263	0.071
Natural childbirth, *n*	115 (75.2%)	396 (76.7%)	0.163	0.686
Meconium delay, *n*	38 (24.8%)	96 (18.6%)	2.861	0.091
Birth asphyxia, *n*	0 (0.0%)	12 (2.3%)	/	/
Premature sepsis, *n*	58 (37.9%)	113 (21.9%)	15.895	0.000
ABO hemolysis, *n*	44 (28.8%)	180 (34.9%)	1.988	0.159
RH hemolysis, *n*	15 (9.8%)	25 (4.8%)	4.318	0.037
G6PD deficiency, *n*	35 (22.9%)	36 (7.0%)	31.445	0.000
Hypoglycemia	10 (6.5%)	12 (2.3%)	6.578	0.010
Infectious, *n*	3 (1.9%)	8 (1.6%)	0.000	0.991
Metabolic acidosis, *n*	4 (2.6%)	9 (1.7%)	0.123	0.725
Cranial hematoma/hemorrhage, *n*	18 (11.8%)	65 (12.6%)	0.075	0.784
Total bilirubin, μmol/l	486.3 ± 88.5	384.2 ± 52.6	17.712	0.000
Direct bilirubin, μmol/l	31.2 ± 18.6	15.4 ± 17.9	9.503	0.000
Indirect bilirubin, μmol/l	455.1 ± 79.6	368.8 ± 46.5	16.802	0.000
Total protein, g/l	55.4 ± 6.1	56.3 ± 5.2	−1.804	0.072
Albumin, g/l	35.1 ± 2.8	34.8 ± 3.8	0.906	0.365
Total bilirubin/albumin, g/l	13.8 ± 3.6	10.6 ± 2.5	12.464	0.000
Hemoglobin, g/l	138.5 ± 35.6	146.5 ± 28.8	2.851	0.004

### Relationship between bilirubin-albumin ratio and bilirubin encephalopathy

We performed a univariate logistic regression analysis with the bilirubin encephalopathy as dependent variable and other factors as independent variables, including gestation age, admission age, gender, birth weight, maternal gestational diabetes, natural children, and clinical parameters. The results from univariate regression indicated that the total bilirubin/albumin ratio was positively associated with bilirubin encephalopathy (odds ratio (OR) = 1.67, 95% confidence interval (CI): 1.59–3.14). The total bilirubin, direct bilirubin, and indirect bilirubin were also related to encephalopathy. The hemoglobin showed a negative relationship with bilirubin encephalopathy. After adjusting some potential cofounding factors, the total bilirubin-albumin was still associated with bilirubin encephalopathy. The higher total bilirubin-albumin ratio increased the risk of bilirubin encephalopathy by 23% (OR = 1.23, 95% CI: 1.16–2.48). Besides, the birth weight, premature sepsis, G6PD deficiency and hypoglycemia, and lower hemoglobin were also associated with increased risk of bilirubin encephalopathy. The details of results are presented in the Table 2.

**Table 2:** Logistic regression about relative factors of bilirubin encephalopathy

Factors	Univariate analysis			Multivariate analysis		
	OR	95% CI		OR	95% CI	
Gestational age, week	1.36	0.67	2.31			
Admission age, h	1.16	1.09	2.06			
Gender (male/female)	1.21	0.83	1.77			
Birth weight (g)	1.29	1.18	2.34	1.59	1.11	2.16
Maternal gestational diabetes, *n*	1.47	0.97	2.24			
Natural childbirth	0.92	0.60	1.40			
Meconium delay	1.45	0.94	2.22			
Premature sepsis	2.18	1.48	3.21	1.23	1.16	3.41
ABO hemolysis	0.75	0.51	1.12			
RH hemolysis	2.13	1.10	4.16			
G6PD deficiency	3.95	2.38	6.57	2.89	1.83	4.12
Hypoglycemia	2.94	1.24	6.94	1.82	1.38	4.61
Infections	1.27	0.33	4.85			
Metabolic acidosis	1.51	0.16	4.98			
Cranial hematoma/hemorrhage	0.93	0.53	1.61			
Total bilirubin	1.26	1.18	2.64	1.21	1.09	2.54
Total protein	1.35	0.87	1.94			
Albumin	1.64	0.39	2.38			
Total bilirubin/albumin	1.67	1.59	3.14	1.23	1.16	2.48
Hemoglobin, g/l	0.54	0.23	0.87	0.36	0.21	0.69

## Discussion

In the present study, we found that there was a positive correlation between bilirubin-albumin ratio level and bilirubin encephalopathy. The bilirubin-albumin ratio could be a potential predictor of bilirubin-albumin ratio in neonates’ patients. The risk increased with the elevated bilirubin-albumin ratio after adjusting for a lot of potential confounding factors.

The early bilirubin metabolism in neonates is characterized by reduced excretion or increased secretion of bilirubin, and the serum bilirubin level is often higher than that of adults, leading to an increasing incidence of hyperbilirubinemia year by year. Currently, the incidence of hyperbilirubinemia among neonates is reported to be from 9.1 to 51% [12]. This disease is involved in many complex clinical factors. According to the bilirubin level and formation reasons, this disease can be divided into current clinical general high neonatal bilirubin levels, pathological and physiological, and the former often hint some basic diseases with different levels of bilirubin brain damage. If timely intervention was not performed, extremely elevated serum bilirubin level may appear and increase bilirubin encephalopathy, resulting in neonatal irreversible neurological damage [13,14]. Therefore, in addition to identifying serum total bilirubin (TSB) level and monitoring TSB dynamics as early as possible, clinical evaluation of bilirubin brain injury in neonates with hyperbilirubinemia is also very important. Some studies have confirmed that the fluctuation range of TSB minimum value in the occurrence of bilirubin encephalopathy is large, and the biliary red encephalopathy can occur when the blood exchange line is lower than that recommended in the guidelines [15,16]. In the present study, it was also found that the peak value of TSB in children with biliary red encephalopathy fluctuated in a wide range when accompanied by different risk factors, which again verified the conclusion that TSB alone could not accurately predict the occurrence of bilirubin encephalopathy. We also found that TSB value has a poor effect on the recalescence of short-term poor prognosis. However, TSB was found to be highly accurate in predicting long-term prognosis (jaundice and long-term hearing impairment). Since there is no follow-up long-term random study in the present study, it is impossible to evaluate whether TSB can be used as an effective indicator to predict long-term prognosis. There has been controversy about whether B/A is more effective than TSB in predicting bilirubin encephalopathy [17,18]. It has been suggested that the sensitivity and specificity of B/A to acute injury caused by bilirubin reach 100% when the broad value of B/A is higher than TSB [19]. However, another study showed that although TSB and B/A were both effective indicators for predicting bilirubin-induced neurological injury, the use of B/A alone was not superior to the use of TSB alone [20]. In our study, B/A fluctuated widely in the presence of etiological risk factors. Both TSB and B/A are close to moderate accuracy in the prediction of hearing impairment, but B/A is not superior to TSB. B/A also plays a poor role in predicting short-term prognosis, and previous studies have shown that B/A is no better than TSBP21 in predicting long-term prognosis of the nervous system [21]. In general, the present study did not find that B/A is better than TSB in predicting the occurrence and prognosis of bilirubin encephalopathy. TSB and B/A, the two laboratory monitoring indicators in the present study, have limited effects on the prediction of bilirubin encephalopathy, prompting us to search for more effective predictors. Bilirubin nerve poison caused mainly by free combination of bilirubin, although TSB and B/A to A certain extent can reflect not combined with the content of bilirubin in the cycle, but one of the many interference factors, such as the molecular environment is not stable, or some competition in the presence of can decrease the rate of binding bilirubin, albumin, made under the condition of invariable TSB and B/A, free elevated bilirubin [22,23]. However, there is still a lack of free bilirubin detection method, which can be widely used in clinical practice [24]. There is also a lack of sufficient clinical research for the initial threshold of its treatment, and further exploration is needed in the future.

There are some limitations for the present study. Frist, one of the study limitations is the study design type, the case–control design is weak in confirming the cause-effect than cohort study. Second, the present study was conducted at a single venue where the quality of clinical evaluation was carefully monitored. Even so, we encountered uncertainties in assigning risk factors for bilirubin encephalopathy. Third, exchange transfusion was associated with worsening bilirubin encephalopathy in several infants, potentially confounding the relationship between admission test values and outcome. Finally, the inability to administer optimal intensive phototherapy and occasional delays in receiving blood for exchange transfusion might have influenced outcomes. Notwithstanding these limitations, the ability of admission serum chemistries to predict thresholds for both acute and post-treatment outcomes was rather remarkable.

The bilirubin-albumin ratio is a useful predictor for bilirubin encephalopathy. Therefore, it is suggested that enough attention should be paid to the clinical practice to actively reduce jaundice in newborns with hyperbilirubinemia and high B/A ratio, and strengthen follow-up to minimize the risk of neurological sequelae.

## Abbreviations

CI: confidence interval
G6PD: glucose 6 phosphate dehydrogenase
MRI: magnetic resonance imaging
OR: odds ratio
TSB: total serum bilirubinemia
